# Mechanism of Mitochondrial Connexin43′s Protection of the Neurovascular Unit under Acute Cerebral Ischemia-Reperfusion Injury

**DOI:** 10.3390/ijms17050679

**Published:** 2016-05-05

**Authors:** Shuai Hou, Ping-Ping Shen, Ming-Ming Zhao, Xiu-Ping Liu, Hong-Yan Xie, Fang Deng, Jia-Chun Feng

**Affiliations:** Department of Neurology and Neuroscience center, the First Hospital of Jilin University, Changchun 130021, China; houshuai0310@163.com (S.H.); blush135@163.com (P.-P.S.); zhaommchn@163.com (M.-M.Z.); liuxiuping12345@163.com (X.-P.L.); xiehy321@163.com (H.-Y.X.); dengfang1212@126.com (F.D.)

**Keywords:** mitochondrial connexin43, mitochondria, cerebral ischemia-reperfusion, mitochondrial ATP-sensitive K^+^ channel, protein kinase C

## Abstract

We observed mitochondrial connexin43 (mtCx43) expression under cerebral ischemia-reperfusion (I/R) injury, analyzed its regulation, and explored its protective mechanisms. Wistar rats were divided into groups based on injections received before middle cerebral artery occlusion (MCAO). Cerebral infarction volume was detected by 2,3,5-triphenyltetrazolim chloride staining, and cell apoptosis was observed by transferase dUTP nick end labeling. We used transmission electron microscopy to observe mitochondrial morphology and determined superoxide dismutase (SOD) activity and malondialdehyde (MDA) content. MtCx43, p-mtCx43, protein kinase C (PKC), and p-PKC expression were detected by Western blot. Compared with those in the IR group, cerebral infarction volumes in the carbenoxolone (CBX) and diazoxide (DZX) groups were obviously smaller, and the apoptosis indices were down-regulated. Mitochondrial morphology was damaged after I/R, especially in the IR and 5-hydroxydecanoic acid (5-HD) groups. Similarly, decreased SOD activity and increased MDA were observed after MCAO; CBX, DZX, and phorbol-12-myristate-13-acetate (PMA) reduced mitochondrial functional injury. Expression of mtCx43 and p-mtCx43 and the p-Cx43/Cx43 ratio were significantly lower in the IR group than in the sham group. These abnormalities were ameliorated by CBX, DZX, and PMA. MtCx43 may protect the neurovascular unit from acute cerebral IR injury via PKC activation induced by mitoKATP channel agonists.

## 1. Introduction

Much research in recent years has focused on the function of gap junctions (GJs) in various tissues and organs, particularly in vertebrates. GJs are necessary for coordinating cell functions by passing electrical current and exchanging chemical signals and energy substrates among heart or nerve cells [1]. Connexin43 (Cx43) is an important member of the family of GJ proteins, is abundantly expressed in the neurological system, and is critical for the normal function [2]. Although most of the functions of Cx43 in cardiac pathophysiology are GJ-specific, recent studies have reported that Cx43 functions outside of intercellular communication [3,4]. In addition to the sarcolemma of cardiomyocytes and brain cells, Connexins are found in the mitochondria, where they take part in the regulation of mitochondrial matrix ion fluxes and respiration [5]. Additionally, Cx43 phosphorylation in cardiomyocyte mitochondria is dramatically modulated during ischemia [6], most likely depending on the content of ATP [7]. However, mitochondrial Cx43 (mtCx43)’s correlation with mitochondrial dysfunction in the neurovascular unit, and whether or not it is involved in the pathogenesis of cerebral ischemic disease remain unclear.

The ATP-sensitive potassium channel (KATP channel) was first discovered by Noma [8] in guinea pig ventricular myocytes in 1983. In addition, Noma hypothesized that this channel participated in the metabolism and membrane electrical activity in myocytes and suggested that opening this channel might serve as a protective mechanism of cardiocytes. Meanwhile, in 1991, Inoue *et al.* [9] first identified the KATP channel in the inner mitochondrial membrane in rats’ liver. Therefore, the KATP channel was divided into the sarcolemmal ATP-sensitive potassium channel (sarcKATP channel) and the mitochondrial ATP-sensitive potassium channel (mitoKATP channel). It is well-known that mitoKATP can provide protective effects for the brain and heart, preserve mitochondrial function [10,11,12], and suppress the overproduction of reactive oxygen species (ROS) during reperfusion, which act as signaling molecules [13,14]. Study results predict a functional interplay between mtCx43 and the mitoKATP channels [15,16]. Thus, we hypothesized that mtCX43 would contribute to neuroprotection via modulation of the mitoKATP channels.

The protein kinase Cs (PKCs) are a family of serine/threonine kinases, which have been shown to regulate cell growth, differentiation, transformation, apoptosis, and tumorigenicity [2,17,18]. The members of the PKC family are grouped into three classes by binding capability: classical PKCs (α, β1, β2, γ), the novel PKCs (δ, ε, θ), and the atypical subgroup (ζ, λ or *i*). In reports characterizing PKC isozymes in the heart, PKC epsilon (PKCε) is described as the major PKC isotype [19]. PKCε, a novel PKC isoform, takes part in the regulation of various cellular functions. Being highly expressed in the brain, several neuroprotective functions, including attenuating oxidative stress [20], have been identified. Additionally, several protein kinases have been indicated in Cx43 phosphorylation, such as mitogen-activated protein kinase (MAPK), PKC, protein kinase A (PKA), and casein kinase [21]. PKC plays a crucial role and is suggested to phosphorylate Cx43 directly at S368 [22]. Additionally, previous studies suggest that the suppression of PKCε by reduced Cx43-pSer368 may be a novel mechanism for mitochondrial dysfunction in heart [23]. We explored the relation between PKC and mtCx43 in cerebral infarction and sought to explain the mechanism.

Thus, the current study focused on the importance of mtCx43 for irreversible brain injury following ischemia-reperfusion (I/R) and its correlation with mitochondrial dysfunction during the pathogenesis of the neurovascular unit to highlight the importance of mtCx43 as an emerging therapeutic target in neuroprotection. In this study, we examined the effect of mtCx43 in animal models of middle cerebral artery occlusion (MCAO). Meanwhile, we used carbenoxolone (CBX) to block the GJ as a positive control group to explore the neuroprotection in different groups [24]. Furthermore, we chose diazoxide (DZX) and 5-hydroxydecanoic acid (5-HD) as the mitoKATP channel agonist and antagonist, respectively, to determine the influence of the channel on mtCx43 or p-mtCx43 expression. Finally, the PKC agonist phorbol-12-myristate-13-acetate (PMA) and the PKC antagonist Ro-31-8425 were injected to determine the connection between mtCx43 and the mitoKATP channel by detecting levels of PKCε and p-PKCε. Our results suggest a novel mechanism where mtCx43 is involved, at least in part, in the pathophysiological process of neuroprotection in cerebral IR injury via the mitoKATP and PKC signaling pathways.

## 2. Results

### 2.1. Cerebral Infarct Volume after MCAO in Different Groups

**Figure 1:**
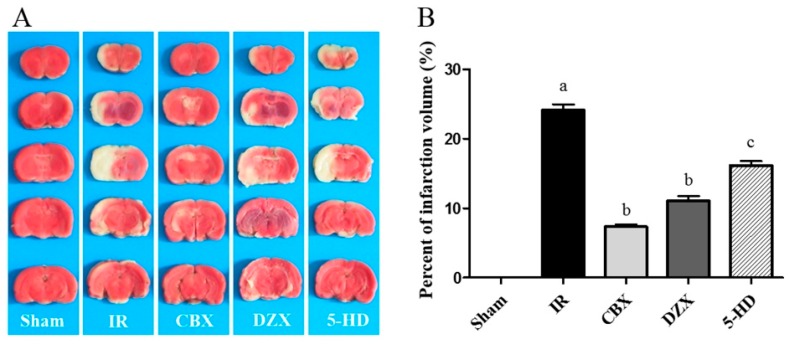
Effect of the mitochondrial ATP-sensitive potassium (mitoKATP) channel on infarction volume in rats with induced middle cerebral artery occlusion (MCAO). (**A**) 2,3,5-triphenyltetrazolim chloride staining of rat brains after 2 h of middle cerebral artery occlusion and 12 h reperfusion; (**B**) The percent of cerebral infarct volume in rats. Data are presented as mean ± standard deviation, *n* = 3 in each group. *F* = 243.3, *p* ≤ 0.05; ^a^ *p* ≤ 0.01 *vs.* Sham; ^b^ *p* ≤ 0.01 *vs.* IR; ^c^ *p* ≤ 0.05 *vs.* DZX. 5-HD: 5-hydroxydecanoic acid; CBX: carbenoxolone; DZX: diazoxide; IR: ischemia-reperfusion.

In order to evaluate the protective effects of the mitoKATP channel in MCAO models in which DZX and 5-HD were injected (described below in Methods), we examined the cerebral infarct volume after stroke. Cerebral infarction volume in rats is shown in Figure 1. Compared with the Sham group, the infarction volume in the IR group was significantly larger (*p* ≤ 0.01). When CBX or DZX was injected 30 min before MCAO, the enlargement of the infarct volume was significantly attenuated. 5-HD significantly reduced the infarct volume attenuation compared with DZX alone (*p* ≤ 0.05). Thus, the activation of mitoKATP could reduce the cerebral infarction volume under I/R injury.

### 2.2. Neurological Deficit Scores after MCAO

**Figure 2:**
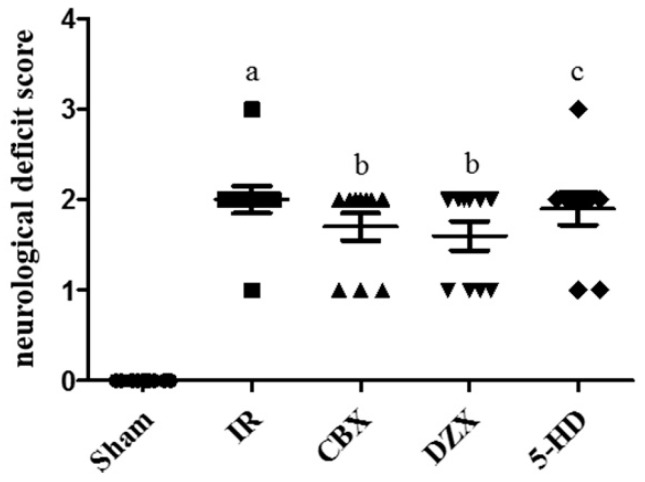
Effect of the mitoKATP channel on neurological deficit scores following middle cerebral artery occlusion in rats. Data are presented as mean ± standard deviation (*n* = 3 in each group). F = 32.22, *p* ≤ 0.05; ^a^ *p* ≤ 0.01 *vs.* Sham; ^b^ *p* > 0.05 *vs.* IR; ^c^ *p* > 0.05 *vs.* DZX. 5-HD: 5-hydroxydecanoic acid; CBX: carbenoxolone; DZX: diazoxide; IR: ischemia-reperfusion.

In addition to the infarction volume, we investigated neurological deficit scores. Rats in the Sham group had a neurological score of 0. Following MCAO, there was a significant deterioration in the neurological deficit scores between the IR group and the sham group (*p* ≤ 0.01). However, no improvement was noted in the scores in the CBX, DZX, or 5-HD groups compared with the IR group after surgery (Figure 2). Thus, CBX and DZX did not improve neurological deficits in rats with cerebral IR injury.

### 2.3. Ultrastructural Damage of the Cell Mitochondria under Transmission Electron Microscopy

**Figure 3:**
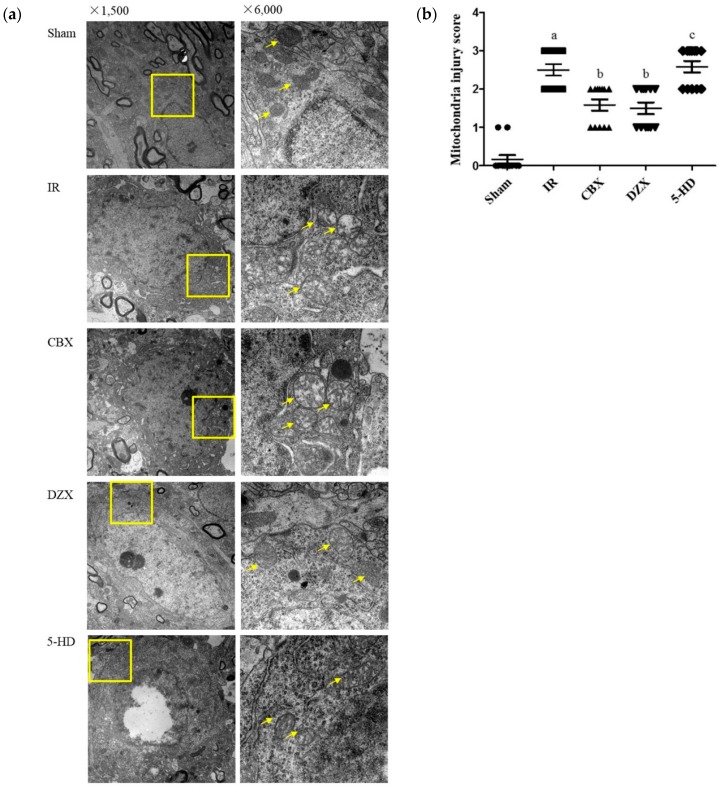
Effect of the mitoKATP channel on mitochondrial ultrastructures in the cortex after I/R injury. (**a**) Mitochondrial ultrastructure in different groups by transmission electron microscopy. The right picture is the zoomed view of the yellow box in the left picture. Mitochondria are indicated with the yellow arrows; (**b**) Scores for mitochondrial injury in different groups. Data are presented as mean ± standard deviation (*n* = 3 in each group). *F* = 46.68, *p* ≤ 0.05; ^a^ *p ≤* 0.01 *vs.* Sham; ^b^ *p ≤* 0.01 *vs.* IR; ^c^ *p ≤* 0.05 *vs.* DZX. 5-HD: 5-hydroxydecanoic acid; CBX: carbenoxolone; DZX: diazoxide; IR: ischemia-reperfusion.

As noted previously, GJ inhibition and mitoKATP channel agonist protected the neurovascular unit from I/R injury. However, their effect on the mitochondria were still unknown. As shown in Figure 3, we examined the mitochondria in the ischemic cortex by transmission electron microscopy (TEM).

In the sham group, the nuclear double-membrane structure was clear, and chromatin was evenly distributed. The mitochondria were circular or oval-shaped and the double membranes were clear, with obvious cristae. In the IR group, the mitochondria were obviously injured; the double membranes were dimmed, the cristae were vague, and vacuolar degeneration was marked. Some mitochondria were even swollen and destroyed. Injuries were slighter in the CBX and DZX groups compared with those in the IR group (*p* ≤ 0.05); the mitochondria were slightly swollen and oval-shaped, while the cristae were partially torn. Meanwhile, mitochondria in the 5-HD group were more obviously injured than those in the DZX group (*p* ≤ 0.05).

### 2.4. Effect of Interference on Nerovascular Unit Apoptosis Following IR Injury

**Figure 4:**
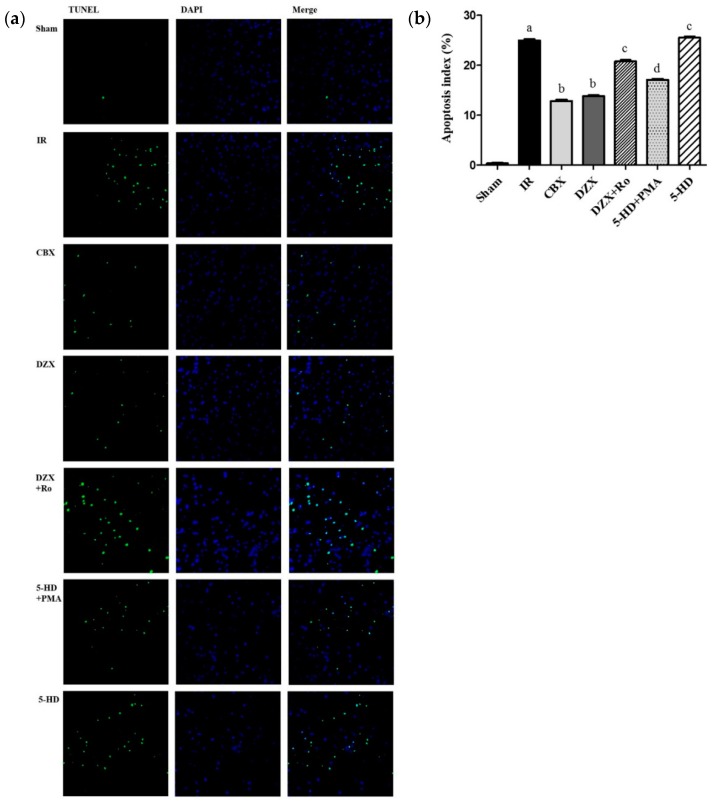
Effect of the mitoKATP channel and protein kinase C on cell apoptosis. (**a**) Representative images of cell apoptosis by TUNEL staining; (**b**) Apoptosic index in different groups. Data are presented as mean ± standard deviation, *n* = 3 in each group. *F* = 975.8, *p ≤* 0.05; ^a^ *p ≤* 0.01 *vs.* Sham; ^b^ *p ≤* 0.01 *vs.* IR; ^c^ *p ≤* 0.05 *vs.* DZX; ^d^ *p ≤* 0.05 *vs.* 5-HD. 5-HD: 5-hydroxydecanoic acid; CBX: carbenoxolone; DAPI: 4′,6-diamidino-2-phenylindole; DZX: diazoxide; IR: ischemia-reperfusion; PMA: phorbol-12-myristate-13-acetate; Ro: Ro-31-8425; TUNEL: transferase dUTP nick end labeling.

As discussed previously, GJ inhibition and mitoKATP channel antagonism could protect the neurovascular unit from I/R injury in morphology and ethology. On the other hand, apoptosis reflected cell viability, and was detected by TUNEL staining and analyzed by the apoptosis index (AI). All positive nuclei were karyopyknotic and stained with green fluorescence. As shown in Figure 4, I/R triggered a significant increase of TUNEL-positive nuclei in the cerebral ischemic cortex compared with those in the sham group (*p ≤* 0.01). CBX and DZX treatment dramatically attenuated apoptosis induced by I/R injury, compared with that in the IR group (*p* ≤ 0.05). However, the reduction of apoptosis was significantly attenuated by 5-HD treatment (*p* ≤ 0.05). Additionally, compared with DZX alone, Ro-31-8425, a PKC inhibitor, induced a greater AI in the ischemic cortex (*p* ≤ 0.05). In contrast, the AI was more markedly reduced in the PMA group than in the 5-HD group (*p* ≤ 0.05). Therefore, we deduced that inhibition of the mitoKATP channel could accelerate the AI under I/R injury, and PKC activation attenuated this increase.

### 2.5. Superoxide Dismutase (SOD) Activity and Malondialdehyde (MDA) Content

**Figure 5:**
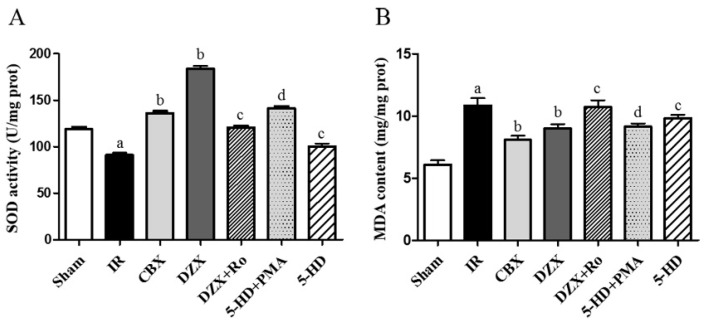
Effects of interference on mitochondrial function in the rat cerebral cortex. (**A**) The SOD activity in different groups; (**B**) The MDA content in different groups. Data are presented as mean ± standard deviation, *n* = 6 in each group; ^a^ *p ≤* 0.05 *vs.* Sham; ^b^ *p ≤* 0.05 *vs.* IR; ^c^ *p ≤* 0.05 *vs.* DZX; ^d^ *p ≤* 0.05 *vs.* 5-HD. 5-HD: 5-hydroxydecanoic acid; CBX: carbenoxolone; DZX: diazoxide; IR: ischemia-reperfusion; MDA: malondialdehyde; PMA: phorbol-12-myristate-13-acetate; Ro: Ro-31-8425; SOD: superoxide dismutase.

Significant amounts of ROS were generated during cerebral I/R injury, which were widely regarded as the initial step in brain damage after stroke. Since SOD and MDA were biomarkers for oxidative stress, their concentrations were examined, and the results are shown in Table 1 and Figure 5.

Compared with the sham group, significantly depressed SOD activity and elevated MDA content were observed in the IR group (*p* ≤ 0.05). Meanwhile, CBX and DZX significantly improved SOD activities and decreased MDA content after ischemia compared with those in the IR group (*p* ≤ 0.05). Conversely, 5-HD and Ro reduced the function of DZX (*p* ≤ 0.05). Furthermore, PMA reversed the damage induced by 5-HD. Thus, activation of the mitoKATP protected mitochondrial function in SOD and MDA via the PKC pathway.

### 2.6. Expression of mtCx43, p-mtCx43, PKCε, and p-PKCε

**Figure 6:**
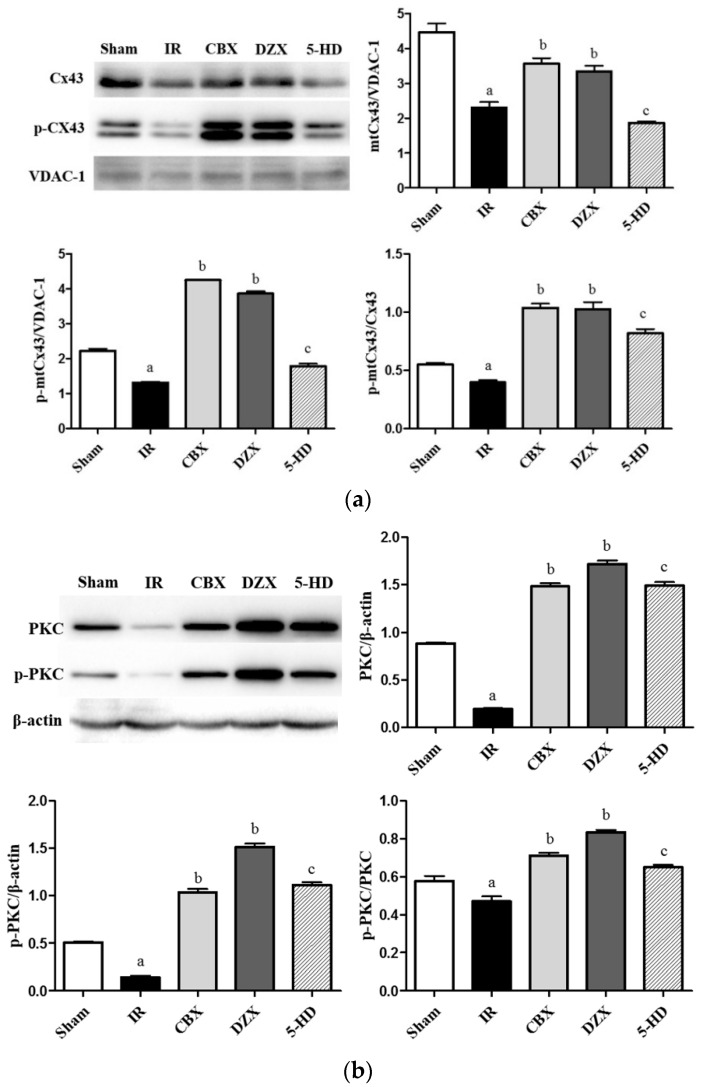
Effects of interference with the mitoKATP channel on the expression of mtCx43, p-mtCx43, protein kinase C epsilon (PKCε), and p-PKCε in the rat cerebral cortex after ischemia-reperfusion injury. (**a**) The expression of mtCx43 and p-mtCx43 in different groups. *F* (mtCx43) = 46.68, *p ≤* 0.05; F (p-mtCx43) = 46.68, *p ≤* 0.05; F (p-mtCx43/Cx43) = 46.68, *p ≤* 0.05; ^a^ *p* ≤ 0.05 *vs.* Sham; ^b^ *p* ≤ 0.05 *vs.* IR; ^c^ *p ≤* 0.05 *vs.* DZX; (**b**) The expression of PKCε and p-PKCε in different groups. Data are presented as mean ± standard deviation, *n* = 6 in each group. F (PKCε) = 470.3, *p ≤* 0.05; F (p-PKCε) = 966.4, *p* ≤ 0.05; F (p-PKCε/PKCε) = 101.9, *p* ≤ 0.05; ^a^ *p* ≤ 0.05 *vs.* Sham; ^b^ *p* ≤ 0.05 *vs.* IR; ^c^ *p* ≤ 0.05 *vs.* DZX. 5-HD: 5-hydroxydecanoic acid; CBX: carbenoxolone; Cx43: connexin43; DZX: diazoxide; IR: ischemia-reperfusion; mtCx43: mitochondrial connexin43; VDAC: voltage-dependent anion channel.

To detect the level of Cx43 in mitochondria, we separated the mitochondria from lysis cells and extracted protein. In mitochondrial proteins, voltage-dependent anion channel (VDAC)-1 was the loading control. As shown in Figure 6, two predominant forms of mtCx43 were detected. The mtCx43/p-mtCx43 ratio was used as a marker of the magnitude of Cx43 phosphorylation. Cerebral I/R injury was associated with a markedly decreased amount of mtCx43 and p-mtCx43 and a reduced mtCx43/p-mtCx43 ratio, compared with those in the Sham group (*p* ≤ 0.05). To elucidate the role of the mitoKATP channel in modulating mtCx43, DZX treatment was used, and the level of mtCx43 was up-regulated compared with that in the IR group (*p* ≤ 0.05). Meanwhile, the enhanced level of mitoKATP channel agonist-related mtCx43 could be reversed to levels similar to those in the I/R injury group after adding the antagonist of 5-HD. The results for PKCε were similar to those for Cx43.

**Figure 7:**
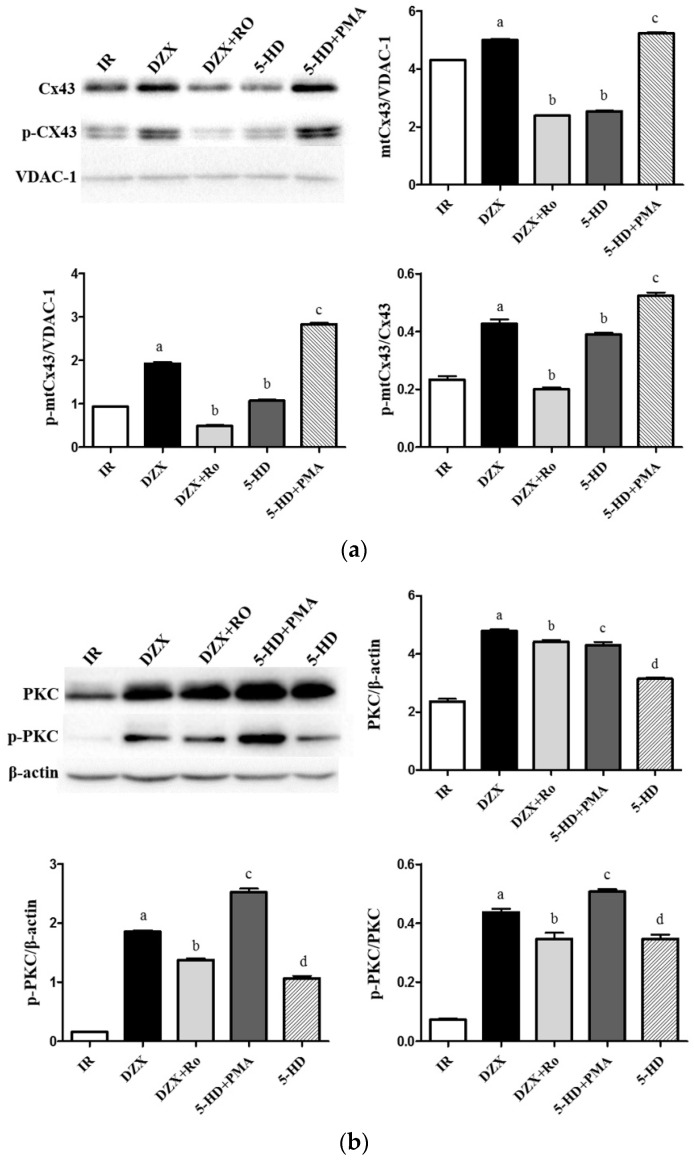
Effects of interference with PKCε on expression of mtCx43, p-mtCx43, PKCε, and p-PKCε in the rat cerebral cortex after ischemia-reperfusion injury. (**a**) The expression of mtCx43 and p-mtCx43 in different groups. Data are presented as mean ± standard deviation, *n* = 6 in each group. *F* (mtCx43) = 3916, *p ≤* 0.05; *F* (p-mtCx43) = 3578, *p ≤* 0.05; *F* (p-mtCx43/Cx43) = 405.5, *p ≤* 0.05; ^a^ *p ≤* 0.05 *vs.* IR group; ^b^ *p ≤* 0.05 *vs.* DZX; ^c^ *p ≤* 0.05 *vs.* 5-HD group; (**b**) The expression of PKCε and p-PKCε in different groups. Data are presented as mean ± standard deviation, *n* = 6 in each group. *F* (PKCε) = 413.3, *p ≤* 0.05; *F* (p-PKCε) = 1499, *p ≤* 0.05; *F* (p-PKCε/PKCε) = 280.1, *p ≤* 0.05; ^a^ *p ≤* 0.05 *vs.* IR; ^b^ *p ≤* 0.05 ^d^ *p ≤* 0.05 *vs.* DZX; ^c^ *p ≤* 0.05 *vs.* 5-HD. 5-HD: 5-hydroxydecanoic acid; Cx43: connexin 43; DZX: diazoxide; IR: ischemia-reperfusion; PMA: phorbol-12-myristate-13-acetate; Ro: Ro-31-8425; VDAC: voltage-dependent anion channel.

Furthermore, as shown in Figure 7, Ro significantly decreased levels of PKCε and mtCx43 compared with DZX alone (*p* ≤ 0.05). Additionally, PMA suppressed the decrease in Cx43 levels that was induced by 5-HD and increased PKCε and p-PKCε expression level, confirming the role of mitoKATP channels in mediating mtCx43 levels via PKC.

## 3. Discussion

**Figure 8:**
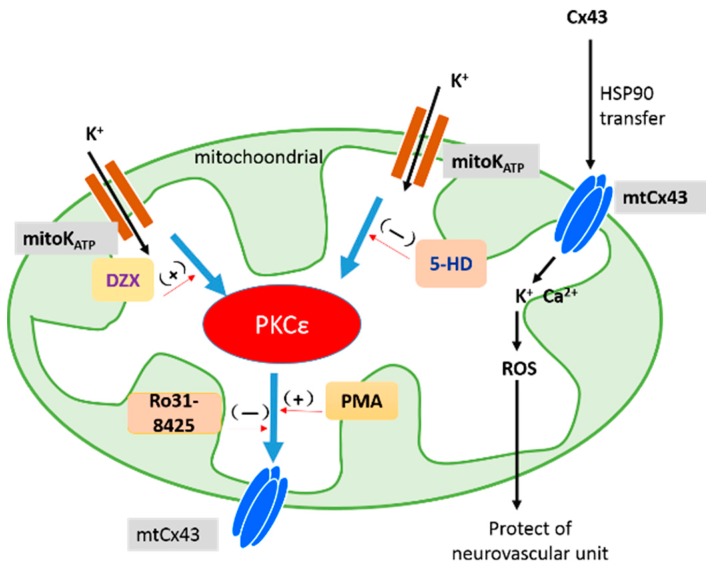
Function and regulation of connexin43 in the mitochondria. 5-HD: 5-hydroxydecanoic acid; Cx43: connexin43; DZX: diazoxide; HSP90: heat shock protein 90; mitoKATP: mitochondrial ATP-sensitive potassium channel; mtCx43: mitochondrial Cx43; PKC: protein kinase C; PMA: phorbol-12-myristate-13-acetate; ROS: reactive oxygen species.

In this study, we showed infarct volume, mitochondrial dysfunction, and decreased mtCx43 expression in cerebral I/R injury, and we also showed that GJ inhibition reduced that damage. Additionally, we observed that all dysfunctions were attenuated by interference with the mitoKATP channel with DZX and 5-HD, suggesting that the mechanisms underlying mitoKATP channel activation that reduced the infarction volume might be related to the attenuated mtCx43 level. Furthermore, the addition of the PKC agonist (PMA) reversed Cx43 phosphorylation, which was attenuated by the mitoKATP channel antagonist, suggesting a pathway among mitoKATP, PKC, and mtCx43. In conclusion, the increases in the level of PKCε by the mitoKATP channel agonist led to up-regulation of Cx43-pSer368 linked with attenuated infarction volume and dysfunctional mitochondria after I/R, as shown in Figure 8. MitoKATP channel agonists may represent a pharmacological target with a clinically safe profile for reducing cerebral I/R injury.

In cerebral I/R injury, the function of Cx43 has become a focus of attention, not only as a GJ but also an unopposed hemichannel at the plasma membrane. Prior studies have documented that connexin expression is affected by several pathophysiological alterations, such as ischemia, diabetes, hypertension, hypercholesterolemia and hypertrophy [25,26]. Furthermore, the channel activities are modified by phosphorylated and de-phosphorylated connexins. In hypoxia, Cx43 is internalized [27] for several minutes and subsequently, the total cellular Cx43 content decreases [28], which is supported by our present study and explains the phenomenon of the decreased total Cx43. Additionally, mtCx43 present in the rat brain in this study was down-regulated in cases of I/R injury. Active mtCx43 appears to inhibit the permeability transition pore, leading to mitochondrial demise and cell death [5]. Thus, mtCx43 is a potential target for reducing the damage of cerebral infarction.

In addition to mtCx43, we also presented evidence for the function of the mitoKATP channel. Activation of this channel preserves mitochondrial function. To evaluate the function of the mitoKATP channel in cerebral infarction, DZX, an agonist of the mitoKATP channel, was selected in this study. Garlid *et al.* [29] first found that the mitoKATP channel was important for cardioprotection in 1997, and DZX opened the mitoKATP channel with a concentration of 0.8 mmol/L, while it only opened the sarcKATP channel at a concentration of 800 mmol/L in the heart. Meanwhile, they observed that diazoxide at low concentrations (5–20 mmol/L) would not open sarcKATP channels and increased the amount of time needed to improve functional recovery in isolated rat hearts subjected to global I/R injury. Subsequent studies [30,31] have also demonstrated that DZX is a selective mitoKATP channel opener and suggested that 5-HD is the channel antagonist. Thus, in this study, we chose DZX at concentrations of 20 mmol/L and 5-HD at concentrations of 100 mmol/L to interfere with the control of mitoKATP channel activation. Wang *et al.* suggested that the opening of astrocytic mitoKATP channels resulted in increased permeability of the mitochondrial GJs for K^+^ influx into the soma and increased electrical coupling [32]. Meanwhile, we found that the brain infarction volume, apoptosis rate, and mitochondrial structure and function were improved by DZX following I/R injury, which was reversed by 5-HD. The results of neurological deficit scores and brain infarction volume were not coincident. In the evaluation of neurological deficit, we found no significance with the injection of interferences. We considered that the ischemic penumbra, which remained viable for several hours in I/R injury, were effective saved by the pharmacologic interventions, but the neurological deficits were recovered slowly. These results suggested that the mitoKATP channel was crucial for neuroprotection against I/R injury. Furthermore, mtCx43 and mtCx43 (p-368) were up-regulated with the help of DZX and down-regulated by 5-HD. We inferred that mitoCx43 was one of the protection targets in cerebral infarction via the mitoKATP channel.

Indeed, we demonstrated that mtCx43 could protect the mitochondria from I/R injury via the mitoKATP channel. However, the connection between these two crucial channels and how they mark is not very clear. At first, we focused on Cx43 as a base point, which is oligomerized in the Golgi/*trans*-Golgi network and transported to the non-junctional plasma membrane through the cytoskeleton [33]. Meanwhile, Cx43 is partly folded by cytosolic heat shock protein 90, presented in specific parts of the translocases of the outer membrane complex, and released into the inner mitochondrial membrane [34]. Cx43 phosphorylation at the S368 active form is phosphorylated by PKC. In our study, we found that PKC and p-PKC levels were increased by DZX and mtCx43 levels were decreased by the antagonist of PKC, which might indicate that PKC is a crucial pathway between the two channels, as shown in Figure 8. We tried to eliminate the protective effect produced by DZX with Ro as a PKCε antagonist and PMA as an agonist to reduce damage by 5-HD. We observed general representative indicators of I/R injury, such as infarction volume, apoptosis rate, and mitochondrial function, as we expected, and that PMA reversed aggravated injury by 5-HD while Ro inhibited the protective effect of DZX. On the other hand, with respect to the mtCx43 level, PMA also proved that PKC had a neuroprotective effect. Waza *et al.* [35] demonstrated the relevance of mitochondrial Cx43 and mitoKATP by co-immunoprecipitation, which provided sufficient evidence for our study. They also considered that mtCx43 was needed for protective signal transduction, including PKCε activation downstream of the DZX-sensitive K_ATP_ channel. However, in Waza *et al.*’s experiment, H9C2 cell hypoxia for 12 h resulted in increased mtCx43, while mtCx43 was down-regulated following hypoxia for 24 h, which was consistent with our results. However, hypoxia for 24 h has not been selected in later experiments. We considered that differences in models resulted in the opposite conclusion and inferred that the opposite might result from the quick metabolism of Cx43 [28]. In our study, the MCAO model was ischemic for 2 h and reperfused for 12 h. Thus, we concluded that PKC activation induced by mitoKATP channel agonist administration contributed to the up-regulation of p-mtCx43 in protecting against injury caused by cerebral infarction. 

Thus, in cerebral ischemia-reperfusion injury, the quantity and functions of Cx43 channel were both regulated. In our study, the expression of mtCx43 and p-mtCx43/mtCx43 was down-regulated, which may have caused by the cell death, protein degradation, and channel dysfunction due to the I/R injury. Meanwhile, expression of mtCx43 was relatively increased by CBX, which maintained stability of the channel structure to assure function, and in the intervention with the mitoKATP channel agonist, mtCx43 level was relatively higher, similar to the result with CBX. However, phosphorylation of Cx43 may lead to the closure of the Cx43 channel. Thus, in I/R injury, phosphorylation of Cx43 is down-regulated and the channel is open, allowing harmful molecules to spread intercellularly. This pathway also explains the protection of CBX, DZX, and PMA. In conclusion, mtCx43 is up-regulated by agonists of the mitoKATP channel via activation of PKC.

These findings propose the mechanism of mtCx43 in its neuroprotective effects, which provide a novel insight into the potential mechanism of protection against cerebral infarction, and is predicted to be a possible target for the treatment of cerebral infarction in clinical studies. Moreover, other pathways are likely involved in this process and future studies should address this possibility.

## 4. Materials and Methods 

### 4.1. Materials

The mitoKATP channel agonist (DZX), antagonist (5-HD), and GJ inhibitor (CBX) were obtained from Sigma (St. Louis, MO, USA); PKC agonist (PMA) and PKC antagonist (Ro-31-8425) were obtained from Millpore (St. Louis, MO, USA); Cx43 (ab11369), p-Cx43 (ab30559), β-actin (ab8227) , GAPDH (ab8245) and VDAC-1 (ab34726) antibodies were obtained from Abcam (Cambridge, MA, USA); PKCε (sc-214) and p-PKCε (sc-12355) were obtained from Santa Cruz Biotechnology (Santa Cruz, CA, USA); horseradish peroxidase-conjugated secondary antibodies were obtained from Bioss (bs-0293G-HRP, bs-0296G-HRP, Beijing, China); 2,3,5-triphenyltetrazolium chloride (TTC) were obtained from Sigma (St. Louis, MO, USA); *In situ* Cell Death Detection Kit was obtained from Roche Molecular Biochemicals (Mannheim, Germany); The SOD activity kit, MDA content kit, functional mitochondria isolation kit and mitochondrial protein extraction kit were obtained from Nanjing Jiancheng Bioengineering Institute (Nanjing, China).

### 4.2. Animals

All experiments were performed with male Wistar rats (weighing 250–280 g) in Jilin University. All animal studies were conducted according to guidelines of the National Regulation of China for Care and Use of Laboratory Animals. This study was approved by the Animals Ethics Committee of the Jilin University of China (10 February 2014, NO.2014-278). The animals were given free access to water and were housed in a temperature-controlled room.

One hundred and thirty-five Wistar rats were randomly divided into seven groups. (1) Rats in the sham group (*n* = 21) were given a lateral cerebral ventricle injection of 0.9% normal saline; (2) Rats in the IR group (*n* = 21) were given a lateral cerebral ventricle injection of 0.9% normal saline 30 min before MCAO; (3) Rats in the CBX group (*n* = 21) were given a lateral cerebral ventricle injection of CBX (5 μg/mL × 10 μL) 30 min before MCAO; (4) Rats in the DZX group (*n* = 21) were given a lateral cerebral ventricle injection of DZX (2 mM × 30 μL) 30 min prior to MCAO; (5) Rats in the 5-HD group (*n* = 21) were given a lateral cerebral ventricle injection of 5-HD (100 mM × 10 μL), and after 10 min, DZX was injected 15 min prior to MCAO; (6) The rats in the DZX + Ro group (*n* = 15) were given a lateral cerebral ventricle injection of DZX, and after 10 min, Ro-31-8425 (400 μg/kg) was injected 15 min prior to MCAO; (7) The rats in the 5-HD + PMA group (*n* = 15) were given an intraperitoneal injection of PMA (200 μg/kg) after the injection of 5-HD and DZX.

### 4.3. MCAO Model

As previously described [36], cerebral ischemia was induced using the MCAO filament model. Briefly, a midline neck incision was made, and then, the external carotid artery (ECA), internal carotid artery (ICA), and common carotid artery (CCA) were exposed. The branches of the ECA were ligated and cut. A filament was introduced into the ICA via the ECA. The tip of the filament was coated with silicon. The filament was inserted about 20 mm. The suture around the ECA was then tightened. The proximal microvascular clip in CCA was removed, and the incision was closed. After awakening, rats were kept in the cage during 120 min of MCAO. The animals were anesthetized again and the filament was removed. During surgery, rats’ body temperatures were maintained at normal values by a heating pad. After awakening, the rats were kept in a cage with free access to food and water.

### 4.4. Neurological Evaluation

Neurological evaluation was performed after reperfusion by evaluators blinded to experimental group following a modified scoring system based on that developed by Longa *et al.* [36] as follows: 0, no deficits; 1, difficulty in fully extending the contralateral forelimb; 2, unable to extend the contralateral forelimb; 3, mild circling to the contralateral side; 4, severe circling; 5, falling to the contralateral side. Higher neurological deficit scores indicated more severe impairment of motor motion injury.

The rats were anesthetized as described above and euthanized by decapitation. The ischemic cerebral cortex was rapidly removed. All procedures were performed on ice. For TEM observation, the removed tissue was preserved in a 2.5% (*v*/*v*) solution of glutaraldehyde. The cerebral cortexes were stored at −80 °C until use for Western blot analysis and the test of SOD activity and MDA content.

### 4.5. TTC Staining

All rat brains were sliced into 2 mm sections. Each slice was incubated for 20 min in a 2% TTC solution at 37 °C and then fixed in 4% paraformaldehyde. Infarct volume was determined with image analysis software (ImageJ; National Institutes of Health, Bethesda, MD, USA) and compared among different treatment groups.

### 4.6. Mitochondria Isolation 

Cerebral cortical mitochondria were isolated by differential centrifugation using a functional mitochondria isolation kit. The freshly removed cerebral cortex tissue was weighed and rapidly placed in ice-cold washing liquid (provided with the kit, Reagent A) to dislodge impurities. It was then crumbled and homogenized in a Glass/Teflon Potter homogenizer with isolating fluid, the homogenate was centrifuged at 800× *g* for 10 min, and the supernatant was retained (the caryon and unsolvable cells were removed). The supernatant was centrifuged at 10,000× *g* for 10 min, and the precipitate was retained. They were then washed once, centrifuged at 12,000× *g* for 10 min, and the precipitates were retained (mitochondrial pellets were isolated; see Supplementary Figure S1 for more information). The isolated mitochondria were conserved in preservative fluid and either stored at −70 °C or used immediately.

### 4.7. Analysis of Mitochondrial Ultrastructure by TEM

Three rats in each group were anesthetized with 10% chloral hydrate and perfused with 4% paraformaldehyde 24 h after MCAO. Ultrathin Cortex slices were obtained (600–800 nm). Ultrathin sections were stained with uranyl acetate and citric acid lead, and observed under a transmission electron microscope (Hitachi, Tokyo, Japan). Injury to the mitochondria was evaluated by Flameng scores [37].

### 4.8. TUNEL Staining in Cortical Neurovascular Unit Apoptosis 

Apoptosis was analyzed by terminal deoxynucleotidyl transferase dUTP nick end labeling (TUNEL) assay using the kit. Briefly, 50 μL TUNEL reaction mixture were added to each sample, and the slides were incubated in the dark for 60 min at 37 °C and then washed three times for 5 min, the slides were incubated with 4′,6-diamidino-2-phenylindole (DAPI) to detect the nuclei, and observed with a fluorescence microscope. Apoptosic index was expressed as the number of TUNEL-positive cells/the total number of cells × 100%.

### 4.9. Detection of SOD Activity and MDA Content

After the behavioral test, rats were immediately sacrificed and the cortex extracted. Tissue samples were homogenized in iced saline. SOD activity and MDA content were measured using commercially available detection kits according to the manufacturers’ instructions. SOD activity was tested with the xanthine oxidase method, and MDA content was tested with the thiobarbituric acid method. The samples were analyzed with a spectrophotometer (BioRad, San Diego, CA, USA).

### 4.10. Western Blot Analysis

The ischemic cerebral cortex tissue and mitochondria in the same set of rats were crumbled and homogenized with lysis buffer (RIPA:NaVO_3_:PMSF:NaF = 92:5:2:1). Proteins were abstracted from the cerebral cortex tissue or mitochondria and then the protein concentrations were assayed. Each sample (50 μg) was loaded on a 12% sodium dodecyl sulfate polyacrylamide gel electrophoresis (SDS-PAGE) apparatus and run for about 90 min. Then, the proteins were electrotransferred to polyvinylidene fluoride (PVDF) membranes. The membranes were blocked with antibodies of Cx43 (1:2000), p-Cx43 (1:2000), PKC (1:200), p-PKC (1:200), VDAC-1 (1:2000), and rabbit polyclonal anti-β-actin antibody (1:500), which were diluted with 5% defatted milk powder dissolved in Tween-20PBS (PBST) overnight in 4 °C. The membranes were washed and then incubated with horseradish peroxidase-conjugated secondary antibody for 1 h. Protein bands were quantified with Quantitation One software (Bio-Rad Laboratories, Hercules, CA, USA). Relative abundance was obtained by normalizing the density of proteins against that of β-actin or VDAC-1.

### 4.11. Statistical Analysis 

All data are expressed as mean ± standard deviation of multiple experiments. ANOVA with Fisher’s Least Significant Difference (LSD) post tests were used to compare multiple groups by SPSS 16.0 software (SPSS, Chicago, IL, USA). Statistical significance was detected at the 0.05 level.

## Figures and Tables

**Table 1:** The activity of SOD and MDA content in different groups.

Group (*n* = 6)	SOD Activity (U/mg Protein)	MDA Content (mmol/mg Protein)
Sham	118.9 ± 2.3	6.1 ± 0.3
IR	91.0 ± 2.5 ^a^	10.8 ± 0.6 ^a^
CBX	136.3 ± 2.2 ^b^	8.1 ± 0.3 ^b^
DZX	183.7 ± 3.1 ^b^	8.9 ± 0.3 ^b^
DZX + Ro	120.5 ± 0.2 ^c^	10.7 ± 0.6 ^c^
5-HD + PMA	141.2 ± 2.3 ^d^	9.1 ± 0.3 ^d^
5-HD	100.3 ± 3.1 ^c^	9.8 ± 0.3 ^c^

*F* (SOD) = 359.1, *p* ≤ 0.05; *F* (MDA) = 41.05, *p* ≤ 0.05; ^a^ *p ≤* 0.05 *vs.* Sham; ^b^ *p ≤* 0.05 *vs.* IR; ^c^ *p ≤* 0.05 *vs.* DZX; ^d^ *p ≤* 0.05 *vs.* 5-HD. Data are presented as mean ± standard deviation, *n* = 6 in each group. 5-HD: 5-hydroxydecanoic acid; CBX: carbenoxolone; DZX: diazoxide; IR: ischemia-reperfusion; MDA: malondialdehyde; PMA: phorbol-12-myristate-13-acetate; Ro: Ro-31-8425; SOD: superoxide dismutase.

## Supplementary Materials

Supplementary materials can be found at http://www.mdpi.com/1422-0067/17/5/679/s1.

## Abbreviations

I/R	ischemia-reperfusion
Cx43	connexin 43
mtCx43	mitochondrial connexin 43
GJ	gap junction
K-ATP	ATP-sensitive potassium channel
mitoKATP	mitochondrial ATP-sensitive potassium channel
sarcKATP	sarcolemmal ATP-sensitive potassium channel
5-HD	5-hydroxydecanoic acid
CBX	carbenoxolone
DZX	diazoxide
PMA	phorbol-12-myristate-13-acetate
Ro	Ro-31-8425
PKA	protein kinase A
PKC	protein kinase C
MAPK	mitogen-activated protein kinase
MCAO	middle cerebral artery occlusion
AI	apoptosis index
DAPI	4′,6-diamidino-2-phenylindole
ROS	reactive oxygen species
SOD	superoxide dismutase
MDA	malondialdehyde
TEM	transmission electron microscopy
TUNEL	transferase dUTP nick end labeling
