# Supplementary material for: Mechanism of Mitochondrial Connexin43′s Protection of the Neurovascular Unit under Acute Cerebral Ischemia-Reperfusion Injury
Source: Int J Mol Sci. 2016 May 5;17(5):679. doi: 10.3390/ijms17050679 (PMC4881505; doi:10.3390/ijms17050679)
Supplement: Supplementary file 1 [file ijms-17-00679-s001.pdf]

Supplementary Materials: Mechanism of Mitochondrial Connexin43's Protection of the Neurovascular Unit under Acute Cerebral Ischemia-Reperfusion Injury

Shuai Hou, Ping-Ping Shen, Ming-Ming Zhao, Xiu-Ping Liu, Hong-Yan Xie, Fang Deng and Jia-Chun Feng

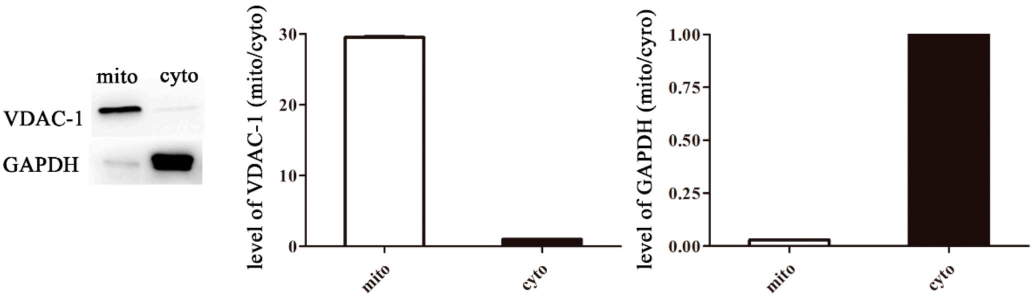

Figure S1. Expression of VDAC-1 and GAPDH in mitochondria and cytoplasm. They are calculated as a percent relative to the level of the cyto group. Data are presented as mean ± standard deviation, n = 6 in each group. mito, mitochondria; cyto, cytoplasm; VDAC, voltage-dependent anion channel.

The VDAC-1 level of the mito group was about 30 times higher than that of the cyto group, which predicted that the isolation of mitochondria were almost intact. The GAPDH level of the cyto group was about 30 times higher than that of the mito group, which predicted that the mitochondria protein were in a high purity.
